# Microarray expression studies on bone marrow of patients with Shwachman-Diamond syndrome in relation to deletion of the long arm of chromosome 20, other chromosome anomalies or normal karyotype

**DOI:** 10.1186/s13039-019-0466-9

**Published:** 2020-01-02

**Authors:** Abdul Waheed Khan, Antonella Minelli, Annalisa Frattini, Giuseppe Montalbano, Alessia Bogni, Marco Fabbri, Giovanni Porta, Francesco Acquati, Rita Maria Pinto, Elena Bergami, Rossella Mura, Anna Pegoraro, Simone Cesaro, Marco Cipolli, Marco Zecca, Cesare Danesino, Franco Locatelli, Emanuela Maserati, Francesco Pasquali, Roberto Valli

**Affiliations:** 1 Genetica Umana e Medica, Dipartimento di Medicina e Chirurgia, Università dell’Insubria, Via J. H. Dunant, 5, 21100 Varese, Italy; 2 Genetica Medica, Fondazione IRCCS Policlinico S. Matteo and Università di Pavia, Pavia, Italy; 3 UOS Milano IRGB, Consiglio Nazionale delle Ricerche, Milano, Italy; 4 European Commission, Joint Research Centre (JRC), Ispra, Italy; 5 Haematopathology Division, European Institute of Oncology, Milano, Italy; 6 Department of Biotechnology and Life Sciences, Università dell’Insubria, Varese, Italy; 7 Department of Pediatric Hematology and Oncology, IRCCS Ospedale Pediatrico Bambino Gesù, Università di Roma Sapienza, Roma, Italy; 8 Pediatric Hematology / Oncology, Fondazione IRCCS Policlinico S. Matteo, Pavia, Italy; 9 SC Oncoematologia Pediatrica, Ospedale Pediatrico Microcitemico “Antonio Cao”, Azienda Ospedaliera Brotzu, Cagliari, Italy; 10 Pediatric Hematology Oncology, Ospedale Donna Bambino, Azienda Ospedaliera Universitaria Integrata, Verona, Italy; 11 Cystic Fibrosis Center, Azienda Ospedaliera Universitaria Integrata di Verona, Verona, Italy

**Keywords:** Shwachman-diamond syndrome, Expression analysis, Clonal chromosome anomalies in bone marrow, *EIF6* gene, Risk of MDS/AML, Somatic genetic rescue

## Abstract

**Background:**

Clonal chromosome changes are often found in the bone marrow (BM) of patients with Shwachman-Diamond syndrome (SDS). The most frequent ones include an isochromosome of the long arm of chromosome 7, i (7)(q10), and an interstitial deletion of the long arm of chromosome 20, del (20)(q). These two imbalances are mechanisms of somatic genetic rescue. The literature offers few expression studies on SDS.

**Results:**

We report the expression analysis of bone marrow (BM) cells of patients with SDS in relation to normal karyotype or to the presence of clonal chromosome anomalies: del (20)(q) (five cases), i (7)(q10) (one case), and other anomalies (two cases). The study was performed using the microarray technique considering the whole transcriptome (WT) and three gene subsets selected as relevant in BM functions. The expression patterns of nine healthy controls and SDS patients with or without chromosome anomalies in the bone marrow showed clear differences.

**Conclusions:**

There is a significant difference between gene expression in the BM of SDS patients and healthy subjects, both at the WT level and in the selected gene sets. The deletion del (20)(q), with the *EIF6* gene consistently lost, even in patients with the smallest losses of material, changes the transcription pattern: a low proportion of abnormal cells led to a pattern similar to SDS patients without acquired anomalies, whereas a high proportion yields a pattern similar to healthy subjects. Hence, the benign prognostic value of del (20)(q). The case of i (7)(q10) showed a transcription pattern similar to healthy subjects, paralleling the positive prognostic role of this anomaly as well.

## Background

Shwachman-Diamond syndrome (SDS) is an autosomal recessive disorder (Online Mendelian Inheritance in Man #260400) characterized by bone marrow failure, peripheral cytopenias and an increased risk of developing myelodysplastic syndrome (MDS) and acute myeloid leukaemia (AML). The patients exhibit several other anomalies, including cognitive impairment [1]. SDS is caused by mutations in the *SBDS* gene in at least 90% of cases [1], but it is genetically heterogeneous. In addition to other functions, the SBDS protein has a pivotal role in ribosome biogenesis [1]. Furthermore, biallelic mutations of two other genes involved in ribosome biogenesis may cause SDS or an SDS-like condition: *DNAJC21* [2, 3] and *EFL1* [4]. Moreover, an SDS-like phenotype may be caused by monoallelic mutations in the *SRP54* gene, which produces a protein that is a key member of the cotranslation protein-targeting pathway [5]. Therefore, SDS may be considered a ribosomopathy.

Clonal chromosome changes are often found in the bone marrow (BM) of patients with SDS. Among them, the most frequent ones include an isochromosome of the long arm of chromosome 7, i (7)(q10), and an interstitial deletion of the long arm of chromosome 20, del (20)(q) [6]. We already postulated that the presence of del (20)(q), with the loss of the *EIF6* gene, results in more efficient ribosome biogenesis and implies both a lower risk of MDS/AML [7] and a milder haematological condition compared to SDS patients without del (20)(q) [8, 9].

The literature offers quite a few expression studies on SDS. Some of them concern the expression of specific genes in *SBDS* knocked-down cell lines (HeLa, NIH3T3) or in BM cells of SDS patients. These studies reveal interesting results; however, the scope of the presented work is limited to a few sets of considered genes [10–12]. We also remark that in some cell lines, such as HeLa, the results of expression analysis might be altered due to high variability of genomic instability and expression profiling among different batches, to the point that some results may be not completely reliable [13]. More extensive expression studies on BM from SDS patients and on other modified cell lines led to the detection of a series of genes that are up- or downregulated. Among those gene sets, many are important in leukaemia pathogenesis or ribosome biogenesis and function [14, 15]. Possible chromosome anomalies were not considered in all those studies.

The benign prognostic role of del (20)(q) that is acquired in BM prompted us to perform an expression study on the BM of patients with del (20)(q) even at the level of the whole transcriptome. In these patients, we report the expression analysis of the *EIF6* gene, of the whole genome, and of gene sets selected as relevant in haematopoiesis, myeloid leukaemias, or myeloid differentiation. These results are compared with those obtained from patients who exhibit other clonal chromosome anomalies or show a normal karyotype in relation to healthy controls.

## Results

Out of the total 17 patients with SDS, chromosome anomalies were found in eight, and their cytogenetic results, at the date of sampling for RNA study, are summarized in Table 1. All patients are identified by their unique patient number (UPN). The clonal del (20)(q) was present in five patients (UPN 6, 13, 20, 68, 85) encompassing the *EIF6* gene in all samples, as demonstrated by array-based comparative genomic hybridization (a-CGH), the i (7)(q10) in one patient (UPN 24) and a clonal unbalanced translocation t(1;16) in one patient (UPN 58). The a-CGH analysis showed that the del (20)(q) in UPN 13 was smaller in the 2017 sample than that in the 2015 sample. One patient (UPN 92), the only one who developed AML, showed clones with complex abnormal karyotypes, with structural anomalies, not better defined, involving chromosomes 1, 2, 3, 5, 8, 10, 11 and 12. Table 1 also provides the percentage of abnormal cells at the date of BM sampling for transcription analysis. These percentages were inferred either from the results of fluorescent in situ hybridization (FISH) on nuclei with informative probes or from the results of a-CGH with the appropriate formula [17] or from chromosome analysis (in one patient).

**Table 1:** Clonal chromosome anomalies in BM, and percentage of abnormal cells at the date of sampling for RNA study

Patient UPN	Sample^a^	anomaly	% abnormal cells
6	2014	del (20)(q11.21q13.13)	44%^e^
13	2015	del (20)(q11.21q13.32)	12%^e^
	2017	del (20)(q11.21q13.13)	52%^f^
20	2013	del (20)(q11.21q13.32)^b^	68%^f^
	2015		60%^f^
	2017		76%^f^
24	2009	i (7)(q10)	30%^f^
58	2014	der(16)t(1;16)(q21;q23)	17%^f^
	2017		15%^f^
68	2016	del (20)(q11.21q13.12) del (20)(q13.12q13.13)^c^	19%^f^
85	2015	del (20)(q11.21q11.23)	14%^f^
	2016		–
	2017		11%^e^
92	2017	complex karyotype^d^	83%^g^

^a^Sample identified by the year of analysis

^b^Presence of an additional subclone with a rearrangement of the del (20)(q), with deleted and duplicated portions of chromosome 20 [16]

^c^Two interstitial deletions with a conserved segment of 2103 Kb in between

^d^Clones with several structural anomalies, not better defined, involving chromosomes 1, 2, 3, 5, 8, 10, 11 and 12

^e^Results of FISH on nuclei

^f^Calculated from a-CGH results

^g^Result of chromosome analysis

In nine patients, no anomalies were present in the BM at the date of sampling for RNA study, according to the available results of chromosome analyses, FISH with probes informative for i (7)(q10) and del (20)(q), and a-CGH (UPN 2, 26, 45, 51, 60, 70, 80, 81, 91). In this paper, we designated these patients as SDS-NK (normal karyotype) patients.

**Fig. 1:**
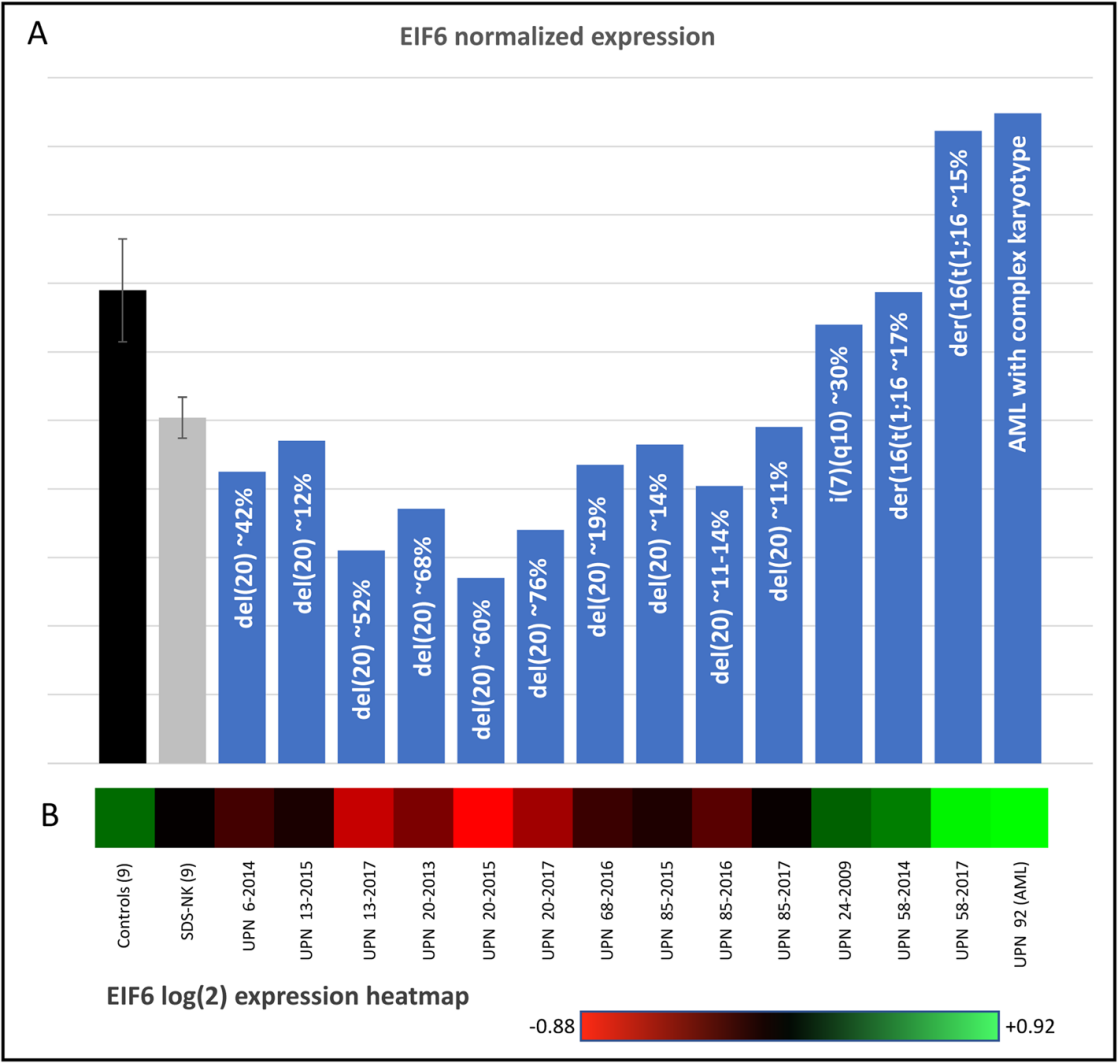
EIF6 expression. **a** *EIF6* normalized expression extrapolated from array raw data. The expression values for the single probe A_23_P210939, included in the array used have been extrapolated by the imported log_2_ raw data with baseline normalization. Values of each specimen has been 2-power exponentially transformed in order to obtain the linear values. **b** The log_2_ heatmap for *EIF6* expression. The patients’ samples are indicated at the bottom and their chromosome anomalies are inserted in the histogram bars, with the percentage of abnormal cells. The black and grey bars refer to the average value of the nine controls and the nine SDS-NK patients; the standard error is indicated

In the context of expression studies, we extrapolated the EIF6 RNA levels from the array raw data, and they are shown in Fig. 1a. The mean and the standard error for the expression levels of the nine normal controls and the nine SDS-NK patients are reported in black and grey bars, respectively, whereas the other bars refer to single patient specimens. Figure 1b shows the log_2_ heatmap for EIF6 expression levels.

**Fig. 2:**
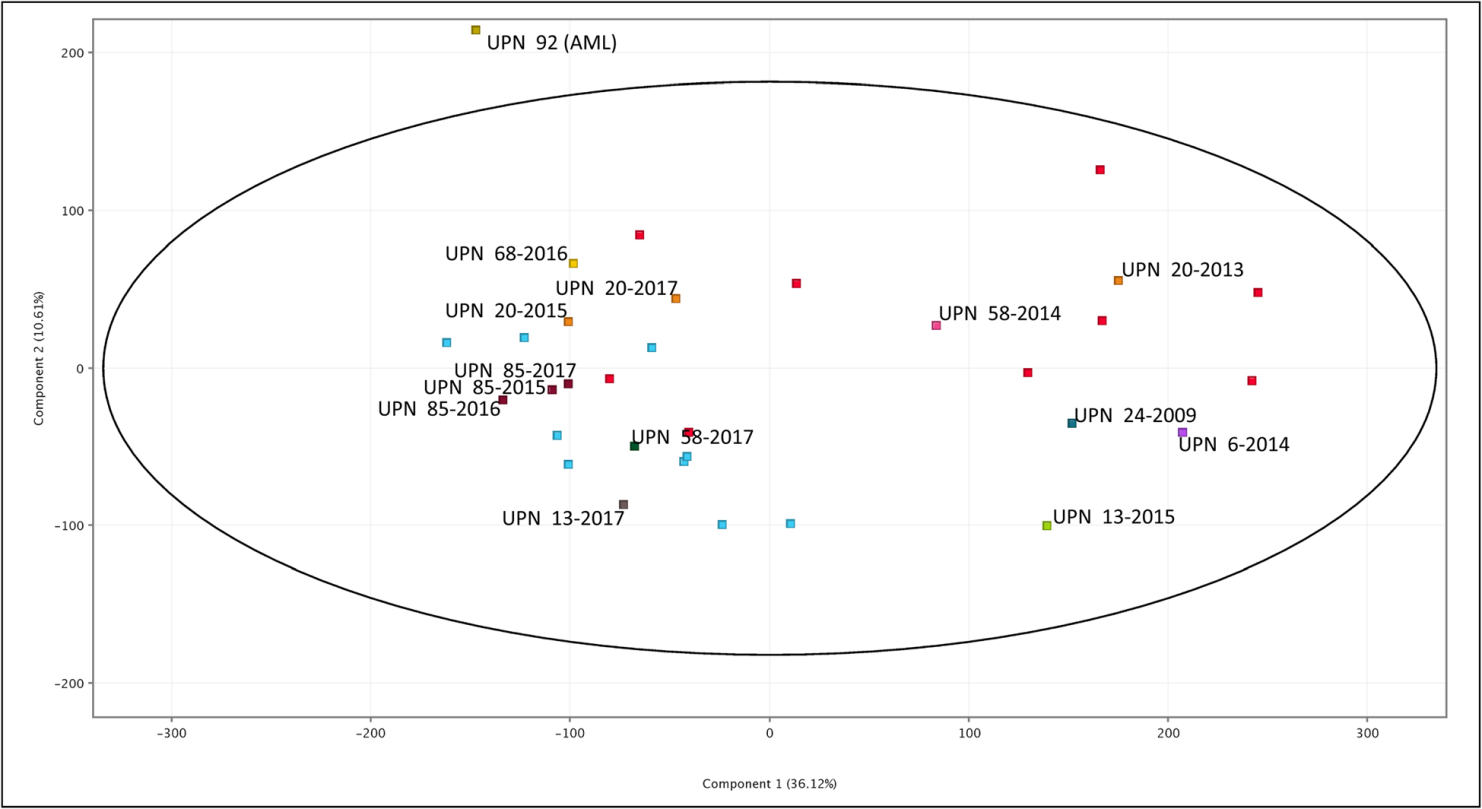
Principal Component Analysis (PCA) plot for the whole transcriptome. Healthy controls are indicated by red squares. SDS-NK patients are indicated by light-blue squares. Patients with chromosomal anomalies are identified by their UPN number and the year of the sample near the related colored squares. Component 1 and 2 percentages of variance are indicated in the two axes. The black ellipse indicates the 95% confidence interval

**Fig. 3:**
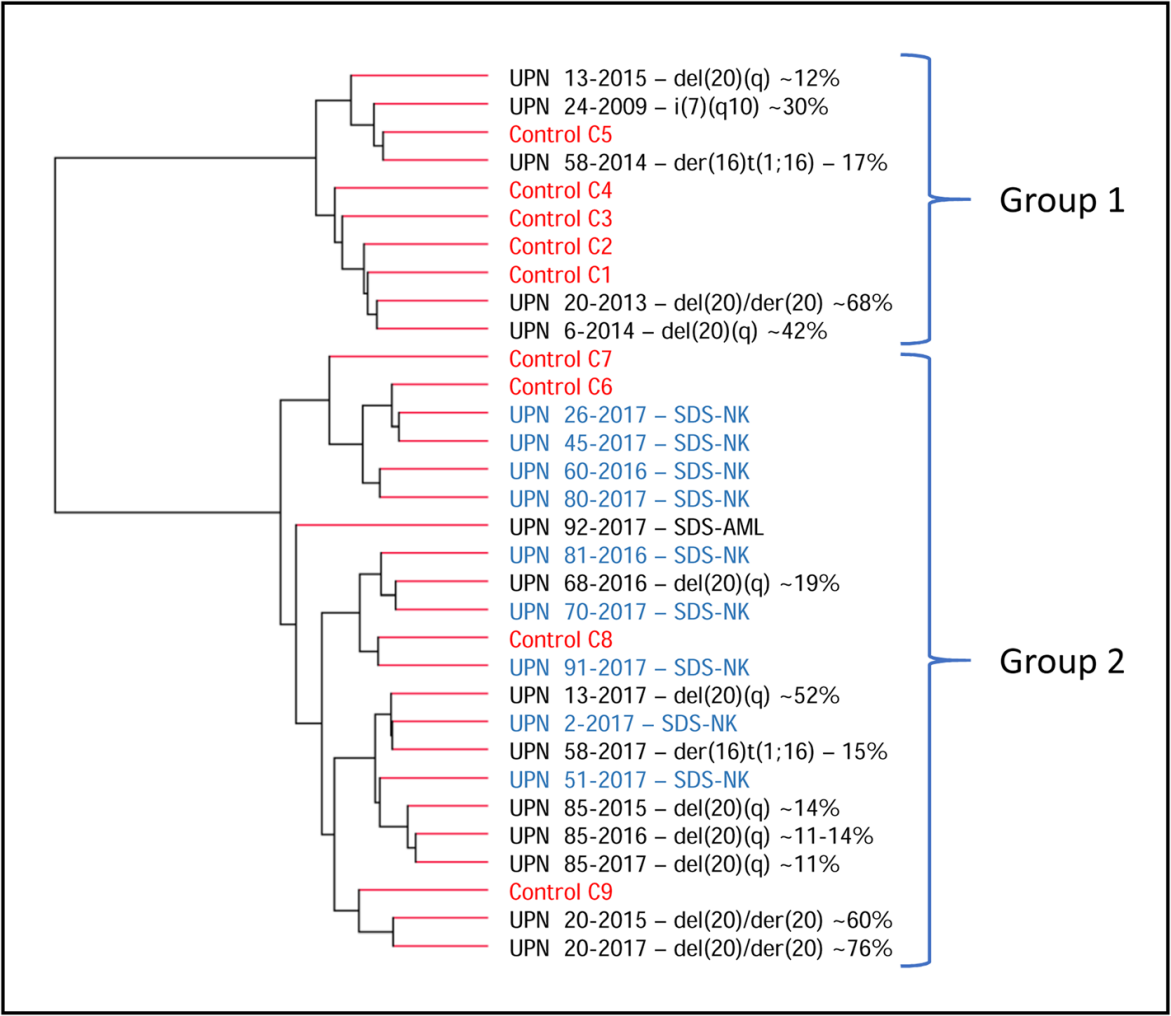
Dendrogram of cluster analysis for the whole transcriptome. Two groups (1 and 2) may be identified. Healthy controls are in red and SDS-NK patients are indicated in light blue with their UPN number. Patients with chromosome anomalies are in black with their UPN number and sample (year), followed by the chromosome anomaly and the percentage of abnormal cells

Whole transcriptome (WT) results were analysed by principal component analysis (PCA) from both SDS patients (with and without chromosome anomalies) and controls. The graph showing PCA for all the subjects under study is shown in Fig. 2. A trend indicating the stratification of patients in groups is appreciable. WT cluster analysis led to the dendrogram shown in Fig. 3.

We analysed some specific gene sets by PCA and cluster analysis (Additional file 1: Figures S1, S2, S3, S4, S5 and S6), chosen as relevant in haemopoiesis and leukaemogenesis:

1. Gene set 1: KEGG Haematopoietic Cell Lineage (map 04640): this pathway is composed of 88 genes, the major portion belonging to cytokines, growth factors and cell differentiation markers that drive the differentiation process of the haematopoietic cell lineage [18].
2. Gene set 2: KEGG Acute Myeloid Leukemia (map 05221): this pathway is composed of 60 genes belonging to oncogenes, protein kinases, tumour suppressor genes, translocation cancer genes and transcription factors that might be deregulated in acute myeloid leukaemia patients [19].
3. Gene set 3: Gene Ontology Myeloid Leukocyte Differentiation (GO:0002573): this pathway is composed of 96 genes that drive a relatively unspecialized myeloid precursor cell to acquire the specialized features of any cell of the myeloid leukocyte lineage [20].

The analysis was performed with the same approach as WT for the three gene sets, and Table 2 summarizes a comparison among the results obtained in the patients with chromosome changes with those of the groups defined by PCA and cluster analyses of WT.

**Table 2:** Transcription study of the selected gene sets relevant in haematopoiesis, leukaemogenesis and myeloid differentiation, identified as 1, 2, and 3 and described in the Results Section: comparison of the results obtained in patients with clonal anomalies (Table 1), grouped here as A and B. Group A includes most healthy controls and Group B all SDS-NK patients. Patient UPN 92, with AML and complex karyotype is not included in the Table, because her expression profile was different from all other subjects investigated and outside the groups identified

Sample^a^	Anomaly – %^b^	Gene Set 1		Gene Set 2		Gene set 3	
		Group		Group		Group	
		A	B	A	B	A	B
UPN 6–2014	del (20) – 44%	•		•		•	
UPN 13–2015	del (20) – 12%		•		•	•	
UPN 13–2017	del (20) – 52%		•		•		•
UPN 20–2013	del (20) – 68%	•		•		•	
UPN 20–2015	del (20) – 60%	•			•		•
UPN 20–2017	del (20) – 76%	•		•		•	
UPN 24–2009	i (7)(q10) – 30%	•		•		•	
UPN 58–2014	der(16)t(1;16) – 17%	•		•		•	
UPN 58–2017	der(16)t(1;16) – 15%		•		•		•
UPN 68–2016	del (20) – 19%	•			•		•
UPN 85–2015	del (20) – 14%		•		•		•
UPN 85–2016	del (20)		•		•		•
UPN 85–2017	del (20) – 11%		•		•		•

^a^See Table 1

^b^Clonal anomaly in short - % abnormal cells

## Discussion

The nine SDS-NK patients showed levels of EIF6 RNA slightly but significantly decreased in comparison to the nine healthy controls (Student’s t test: *p* = 0.02). All patients carrying the del (20)(q) showed a more remarkable decrease compared to the healthy controls, with a trend related to the proportion of cells containing the deletion (Table 1, Fig. 1a). We postulate that low RNA levels lead to decreased amounts of EIF6 protein, even if we did not have enough material to prove it. The patient UPN 24, carrying i (7)(q10), exhibits normal EIF6 levels as expected, as does UPN 58 (with another different chromosome anomaly) (Fig. 1a). The patient with AML and a complex karyotype, UPN 92, exhibited increased levels of EIF6 (Fig. 1a); it is worth noting that numerous studies have demonstrated highly aberrant overexpression of EIF6 in human cancer [21].

In the WT study, the stratification of SDS patients shown by PCA offers some relevant conclusions. In particular (Fig. 2), the SDS-NK patients (light blue squares) group on the left, while the controls (red squares) are more dispersed, and most of them are far from the SDS-NK group. We recall that we worked on RNA extracted from whole marrow samples containing heterogeneous populations of cells; this may explain the lack of strictly homogeneous results in controls. The result, however, indicates that the WT expression pattern of these two groups is truly different. The difference from controls is in agreement with data already reported, but these reports were limited to leukaemia-related genes [14], apoptosis-related genes [10], ribosome biogenesis and RNA processing genes, and other specific genes relevant for SDS phenotype [11, 12, 15] without any relation to the presence of clonal chromosome anomalies.

The patients carrying the del (20)(q), which encompasses the *EIF6* gene in all cases, are indicated in Fig. 2 by squares of other colours, and they are distributed in the plot partially in agreement with the different percentage of cells of the abnormal clone.

Among these patients, the percentage of abnormal BM cells of UPN 68 and UPN 85 was rather low (Table 1), EIF6 expression was only slightly reduced (Fig. 1a), and the PCA plots these BM samples were near the SDS-NK group. Therefore, these two patients with a small number of cells with del (20)(q) show a WT expression pattern similar to SDS-NK patients.

In contrast, patients UPN 6 and UPN 20 (sample 2013), who carry a high proportion of cells with del (20)(q) in the BM (Table 1), with evident decreased levels of EIF6 transcript (Fig. 1a), are plotted in the PCA graph rather distantly from SDS-NK patients. The other two specimens of UPN 20 (sampled in 2015 and 2017, with similar del (20)(q) cell proportions and *EIF6* hypoexpression patterns) are plotted closer to the SDS-NK group. This patient also carried a subclone with a further rearrangement of the del (20)(q), with deletion of the short arm and portions of the chromosome duplicated and deleted [16]. The proportion of this subclone increased from 2013 to 2017, while neutropenia worsened: the difference in expression might be due to this subclone. We postulate that the loss of EIF6 protein was enough to give a transcription pattern similar to controls in 2013 but was less effective in 2015 and 2017. This could explain the different plots of the sample UPN 20–2013 from UPN 20–2015 and UPN 20–2017. The patient UPN 13 exhibited an unexpected pattern for the two specimens from 2013 and 2015. In particular, the UPN 13–2015 sample has a low number of cells with del (20)(q) (Table 1), and *EIF6* expression is only slightly reduced (Fig. 1a). It is plotted in the PCA far from the SDS-NK group. In contrast, specimen UPN 13–2013, with a high proportion of cells with del (20)(q) and a remarkably low level of *EIF6* transcript, is plotted closer to the SDS-NK group. In fact, patient UPN 13 showed two different extents of the deletion in these two different specimens (Table 1). This could explain the differences in the PCA plots.

In general, these data indicate that patients with a high proportion of cells containing del (20)(q) show a WT expression pattern similar to healthy controls in the absence of further changes that may modify the pattern. The positive prognostic role of del (20)(q) would be a consequence of this type of rescue mechanism [8, 9], although it would be limited to cases with a high proportion of abnormal cells [22].

Patient UPN 24, with the i (7)(q) present in ~ 30% of the cells (Table 1), is plotted by the PCA algorithm far from the SDS-NK group. In the isochromosome, the *SBDS* gene is present twice in the form of the mild mutation 258 + 2 T > C, and this fact leads to a different form of rescue mechanism in ribosome biogenesis, impaired by *SBDS* mutations, thanks to some amount of normal SBDS protein [23]. UPN 58, with specimens in 2014 and 2017, carries an unbalanced complex rearrangement that involves chromosomes 1 and 16 (Table 1). The two samples of this patient are plotted differently in the graph. We have no clear-cut explanation for this result, but in conditions different from SDS, gene effects of unbalanced chromosome anomalies may be detected and cause specific pathologic features [24]. The only patient that developed AML (UPN 92) has a complex karyotype (Table 1) and is plotted in the PCA graph far from all the other patients and outside the 95% confidence interval (Fig. 2).

The dendrogram shown in Fig. 3 resembles the PCA plots of Fig. 2. The interconnection lines identify two groups (1 and 2) with similar distribution to the PCA plot commented above.

The transcription study of the selected groups of genes relevant in haematopoiesis, leukaemogenesis and myeloid differentiation defined in the Results section gave results in PCA largely similar to WT: SDS-NK constitute a well-defined group in all gene sets, while most healthy controls do not constitute a real group and are more dispersed in the plot (Additional file 1: Figures S1, S3 and S5). Cluster analysis based on dendrogram diagrams and related heatmaps confirmed this difference, with particular evidence for gene sets 1 and 2 (Additional file 1: Figures S2, S4 and S6).

Regarding patients carrying clonal chromosome changes, Table 2 shows a comparison of their results with healthy donors and SDS-NK patients. Most patients carrying del (20)(q) at low percentages fall in the group of SDS-NK patients for all gene sets (group B in Table 2), which is expected because EIF6 RNA in these patients is close to normal levels and cannot lead to a rescue of the altered *SBDS* pathway. On the other hand, most of the patients with higher percentages of del (20)(q) fall closer to healthy controls (group A in Table 2), as expected by the rescue mechanism postulated when the level of EIF6 is reduced. Few exceptions are present, and the explanation would be as for WT. Additionally, the only patient with i (7)(q10) falls in the group of the healthy controls, as expected, by the other rescue mechanism described [23].

The following points about the three gene sets analysed are worth highlighting.

- Gene set 1: An interesting subset of genes, including the oncogene *KIT*, *THPO* (Thrombopoietin), *EPO* (erythropoietin), *GP1BA* (Glycoprotein 1b Platelet Subunit Alpha), and some cytokines, are upregulated in controls and downregulated in SDS-NK patients (Additional file 1: Figure S2). Another group involving many cluster differentiation (CD) genes and other cytokines is upregulated in the SDS-NK group and downregulated in controls.
- Gene set 2: The cluster analysis (Additional file 1: Figure S4) firmly indicates a group of genes, including oncogenes and transcription factors, that are upregulated in controls and downregulated in SDS-NK.
- Gene set 3: The cluster analysis also showed that the gene *ANXA2* is extremely downregulated in the healthy controls, while it is expressed within the baseline level in the SDS-NK group (Additional file 1: Figure S6). *ANXA2* is frequently upregulated in many types of cancers [25]. A group of genes (*IL31RA*, *TNFSF11*, *TNFSF11A*, *KIT*, *CSF1*, *CSF2*, *CSF3*, *IL25*, *GPC3*, *FARP2*, *EFNA2, EPHA2*, *BMP4*, *CASP10*) is upregulated in healthy controls and, interestingly, in UPN 6, UPN 13–2015, UPN 20–2013, with del (20)(q), in UPN 24, with i (7)(q10), and in UPN 58–2014, with the der (16)(t(1;16). These genes are transcription factors, oncogenes, cytokines, signal transduction genes, growing factors and apoptotic regulators; they play an important role in many biological systems, including leukocyte differentiation, bone morphogenesis, and macrophage differentiation.

## Conclusions

In summary, our transcription study shows the following:

- There is a difference between gene expression in BM of SDS patients and healthy subjects, both at the level of WT and that of selected gene sets relevant for BM functions;
- In SDS patients, the presence of clonal chromosome anomalies also makes the difference at the transcription level;
- The deletion del (20)(q), with the loss of EIF6 gene, present even in the smallest deletions, changes the transcription pattern of BM: a low proportion of abnormal cells led to a pattern similar to SDS patients without acquired chromosome anomalies, whereas a high proportion exhibit a pattern similar to healthy subjects; hence, the benign prognostic value of the del (20)(q) which has already been demonstrated in many patients [8];
- The single case of i (7)(q10) included in this study showed a benign transcription pattern, similar to healthy subjects, paralleling the already established positive prognostic role of this anomaly as well;
- Too little is known about other acquired clonal anomalies to reach any relevant conclusions for prognosis.

## Methods

### Patient selection and sample preparation

The materials for our study consisted of 23 BM samples from 17 patients with SDS, as in four cases the analysis was repeated at two different dates (two cases) or three (two other cases). The patients included three females and 14 males, with an age range of 2–44 years at the time of sampling for RNA analysis. All patients are part of the cohort of 97 Italian patients who have been followed for cytogenetics since 1999. All patients had biallelic mutations in the *SBDS* gene, including 14/17 cases with the two most frequent mutations. Some analyses were repeated at different dates in subsequent years, as the proportion of abnormal cells may vary considerably in time. A portion of the cytogenetic results has already been reported [16, 22, 26–28]. Table 1 gives the years of the cytogenetic analyses performed at the time of sampling for expression studies. All patients are identified by their UPN, as in our previous publications. We reported and discussed the haematological parameters of the patients with del (20)(q) [8], although the sampling date is often not the same as the present RNA study. Some additional haematological data of all the SDS patients reported here are provided in Additional file 2: Table S1.

Nine healthy subjects were used as controls, and their BM was drawn because they were donors for haematopoietic stem cell transplantation (HSCT).

Informed consent for this study was obtained according to the principles of the Declaration of Helsinki from the patients or the patients’ parents.

Chromosome analyses were performed on BM with routine methods. FISH on BM nuclei was carried out by standard techniques with the following bac probes, informative for the deletion del (20)(q) detected in each patient: RP11-17F3 (UPN 6, 13, 20), CTD-2559C9 (UPN 13), XL Del(20q) probe (Metasystems, Altlussheim, Germany) (UPN 68), RP11-17F3 + RP11-29E13 (UPN 85).

The a-CGH was performed on DNA from BM samples with the 244 K genome-wide system (Agilent Technologies Inc., Santa Clara, CA, USA) according to the manufacturer’s instructions, as already described [29]. All DNA was extracted from BM using a liquid-based Flexigene kit (Qiagen, Hilden, Germany) as recommended by Nacheva et al., 2017 [30].

For expression analysis of patients with SDS and controls, 2 ml of BM material was immediately pipetted into a PAXgene Bone Marrow RNA Tube (Qiagen, Hilden, Germany). The extraction was performed with the PAXgene Bone Marrow RNA Kit (Qiagen, Hilden, Germany). RNA integrity was assessed by Agilent’s Bioanalyzer 2100 instrument (Agilent Technologies, Santa Clara, USA) according to the manufacturer’s instructions. All the RNA samples used in this study exhibited an RNA Integrity Number (RIN) [31] above 8.0.

### Whole transcriptome microarray and bioinformatical analysis

We used the Agilent Microarray System (Agilent Technologies, Santa Clara, USA) to perform microarray expression profiling according to Agilent’s One-Color Microarray-Based Gene Expression Analysis Low Input Quick Amp Labelling Protocol (Version 6.9.1) with Agilent’s Whole Transcriptome (WT) Oligo Human Microarray slides 8 × 60 K format (G4851A, AMADID #028004).

Data analysis was performed using Agilent GeneSpring 14.9.1 software. Data from each sample were imported into the software with the following parameters: Threshold: 1, Logbase: 2, Normalization: Shift to 75.0 percentile, Baseline Transformation: median of all samples.

Clustering analysis was performed by hierarchical analysis on normalized intensity values with Euclidean Distance Metrics and Ward’s linkage rules both on all genes as well as on selected gene sets. PCA was performed by the internal software plugin both with all genes as well as on selected gene sets.

## Supplementary information

**Additional file 1: Figures S1, S2, S3, S4, S5 and S6.** PCA and cluster analysis, with heatmaps and dendrograms, of the three gene sets chosen as relevant in haemopoiesis and leukaemogenesis and defined in the Results section.

**Additional file 2: Table S1.** Blood count and bone marrow cellularity of all the SDS patients here reported at the date of sampling for RNA expression study.

## Data Availability

The data used and analyzed in the current study are available from the corresponding author on reasonable request.

## Abbreviations

a-CGH: Array-based comparative genomic hybridization
AML: Acute myeloid leukaemia
BM: Bone marrow
DNA: Deoxyribonucleic acid
FISH: Fluorescent in situ hybridization
HSCT: Haematopoietic stem cell transplantation
MDS: Myelodysplastic syndrome
PCA: Principal component analysis
RIN: RNA integrity number
RNA: Ribonucleic acid
SDS: Shwachman Diamond syndrome
SDS-NK: SDS with normal karyotype
UPN: Unique patient number
WT: Whole transcriptome
