# Supplementary material for: Microarray expression studies on bone marrow of patients with Shwachman-Diamond syndrome in relation to deletion of the long arm of chromosome 20, other chromosome anomalies or normal karyotype
Source: Mol Cytogenet. 2020 Jan 2;13:1. doi: 10.1186/s13039-019-0466-9 (PMC6941278; doi:10.1186/s13039-019-0466-9)
Supplement: Supplementary file 1 — Additional file 1: Figures S1, S2, S3, S4, S5 and S6. PCA and cluster analysis, with heatmaps and dendrograms, of the three gene sets chosen as relevant in haemopoiesis and leukaemogenesis and defined in the Results section. [file 13039_2019_466_MOESM1_ESM.pdf]

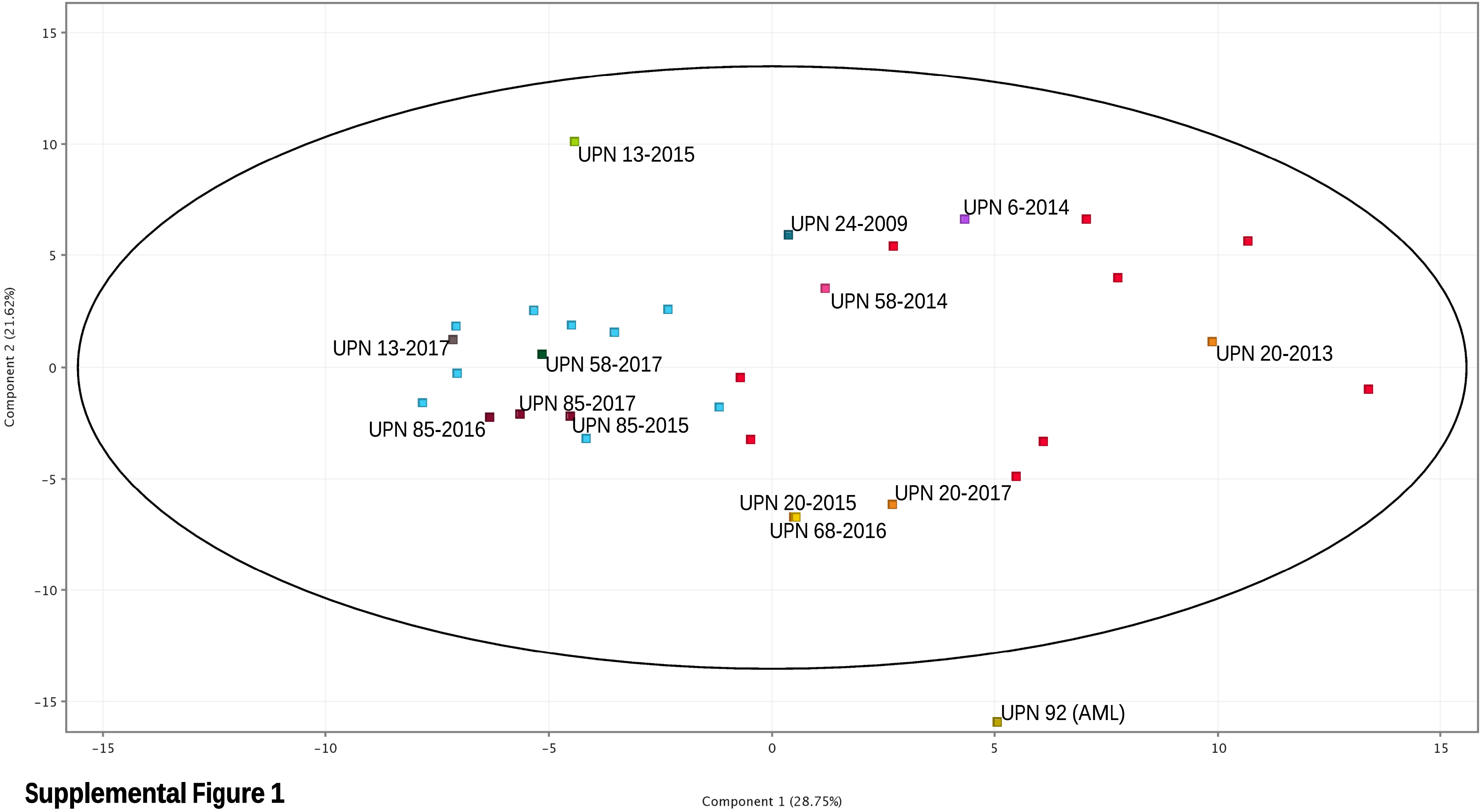

Supplemental Figure 1

Supplemental Fig. 1 Principal Component Analysis (PCA) plot for the Gene set 1 (KEGG Hematopoietic cell lineage). Healthy controls, SDS-NK patients, and patients with chromosome anomalies are identified as in Fig. 2 and in the text. Component 1 and 2 are indicated in the plot. The 95% confidence interval is shown by a black ellipse.

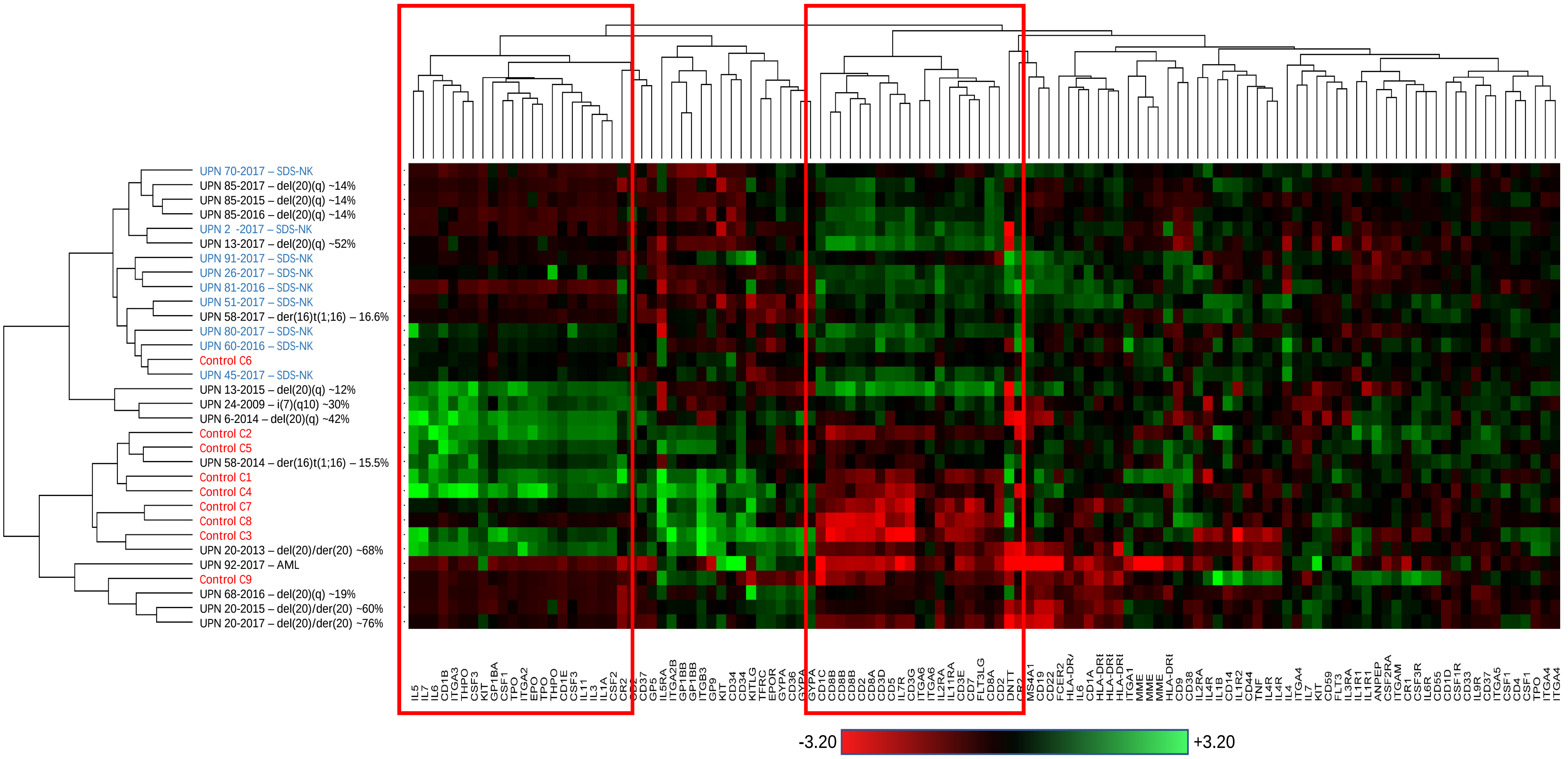

Supplemental Figure 2: KEGG Hematopoietic Cell Lineage

Supplemental Fig. 2 Heatmap and dendrogram of cluster analysis on both the axes for the Gene set 1 (KEGG Hematopoietic Cell Lineage). Healthy controls, SDS-NK and patients with chromosome anomalies are indicated near the dendrogram lines, with evidence of the sample (year), and the karyotype, as in Fig. 3 in the text. Two red boxes put in evidence genes with relevant differences of expression, related to the subgroups of controls and SDS-NK. Gene names and log_2 color bar are under the heatmap.

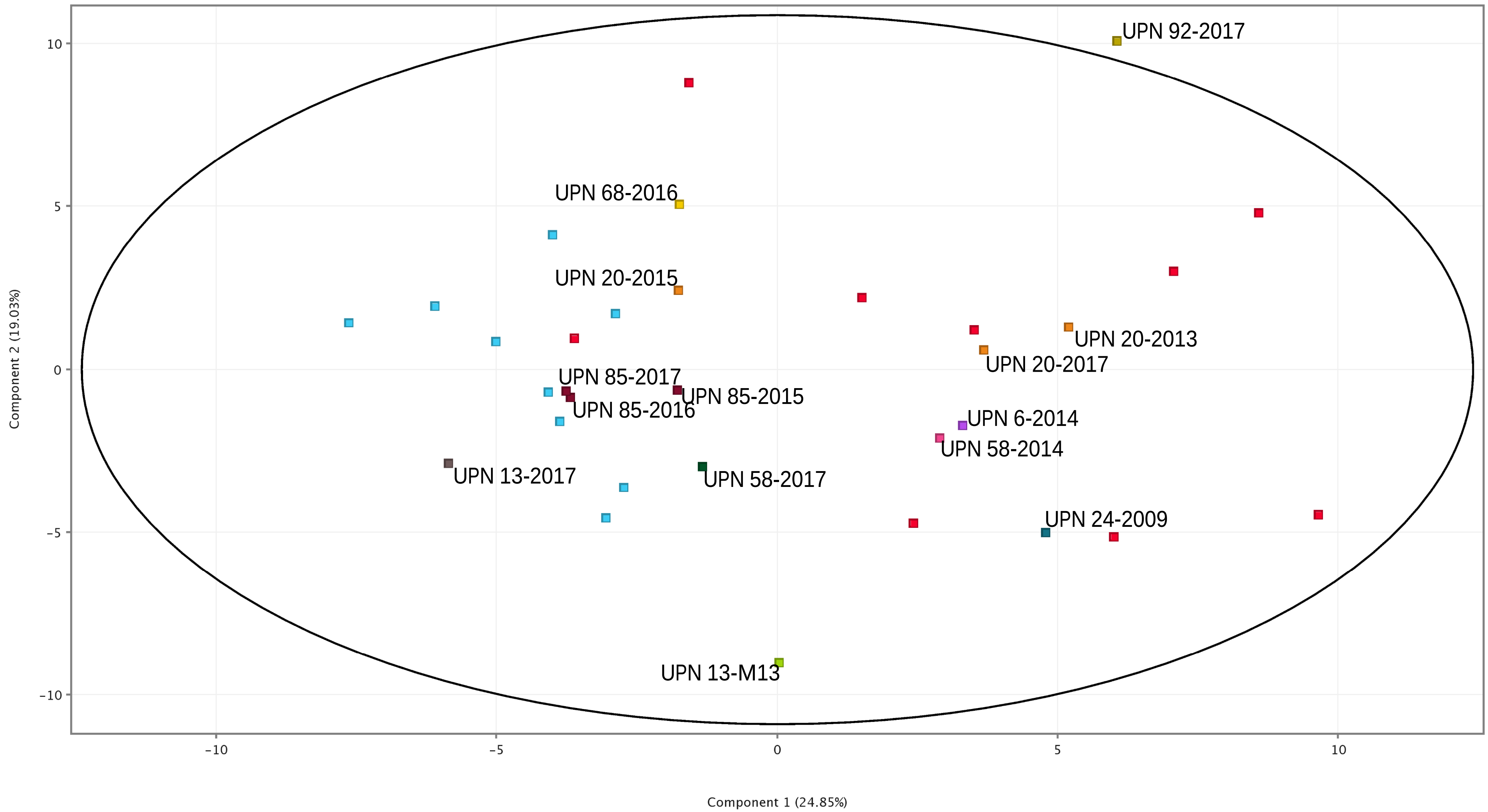

Supplemental Figure 3: KEGG Acute Myeloid Leukemia

Supplemental Fig. 3 Principal Component Analysis (PCA) plot for the Gene set 2 (KEGG Acute myeloid leukaemia). Healthy controls, SDS-NK patients, and patients with chromosome anomalies are identified as in Fig. 2 and in the text. Component 1 and 2 are indicated in the plot. The 95% confidence interval is shown by a black ellipse.

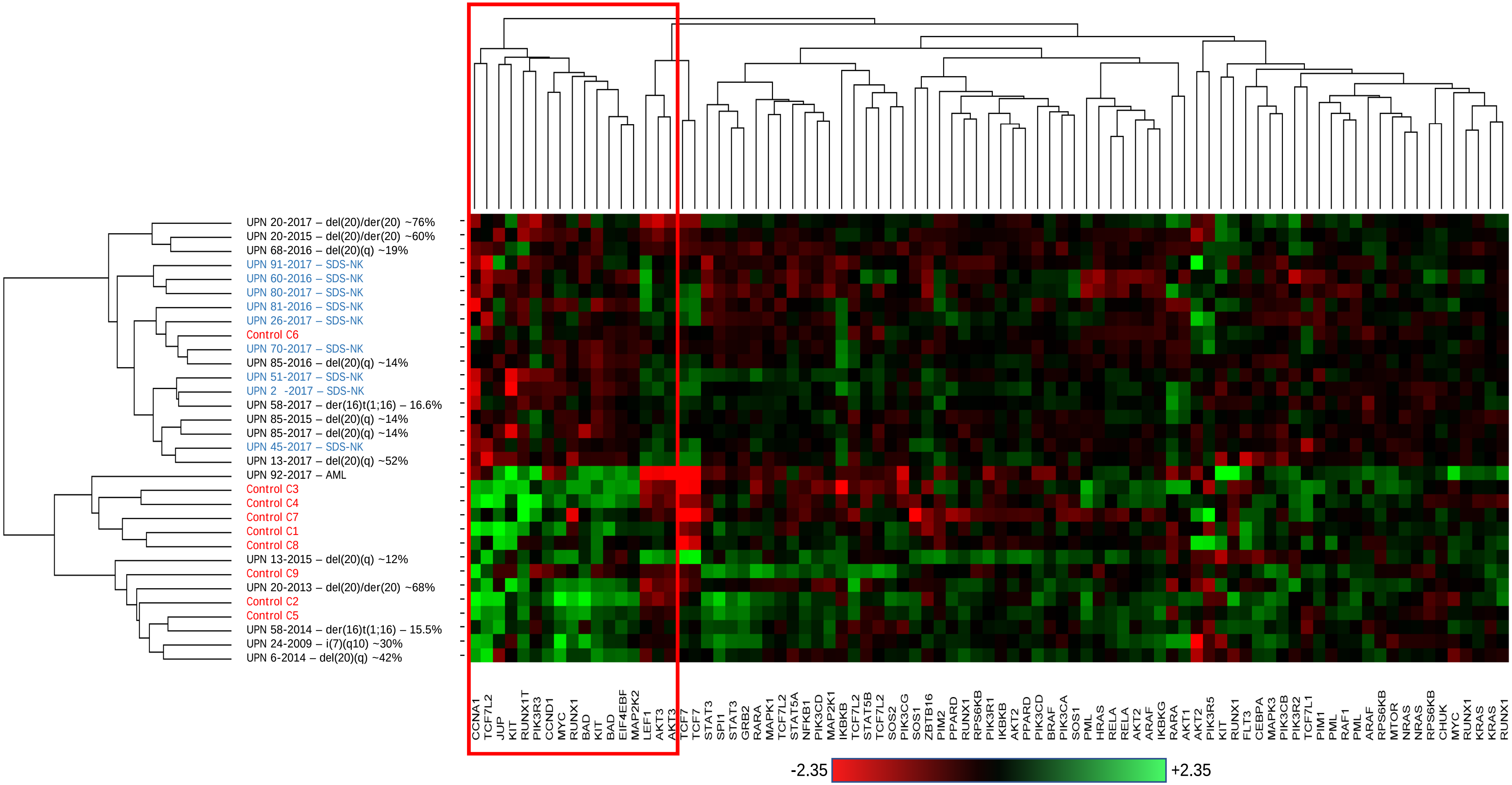

Supplemental Figure 4: KEGG Acute Myeloid Leukemia

Supplemental Fig. 4 Heatmap and dendrogram of cluster analysis on both the axes for the Gene set 2 (KEGG Acute myeloid leukaemia). Healthy controls, SDS-NK and patients with chromosome anomalies are indicated near the dendrogram lines, with evidence of the sample (year), and the karyotype, as in Fig. 3 in the text. A red box put in evidence genes particularly up/down-regulated, with difference in the groups of controls and SDS-NK. Gene names and $\log_2$ color bar are under the heatmap.

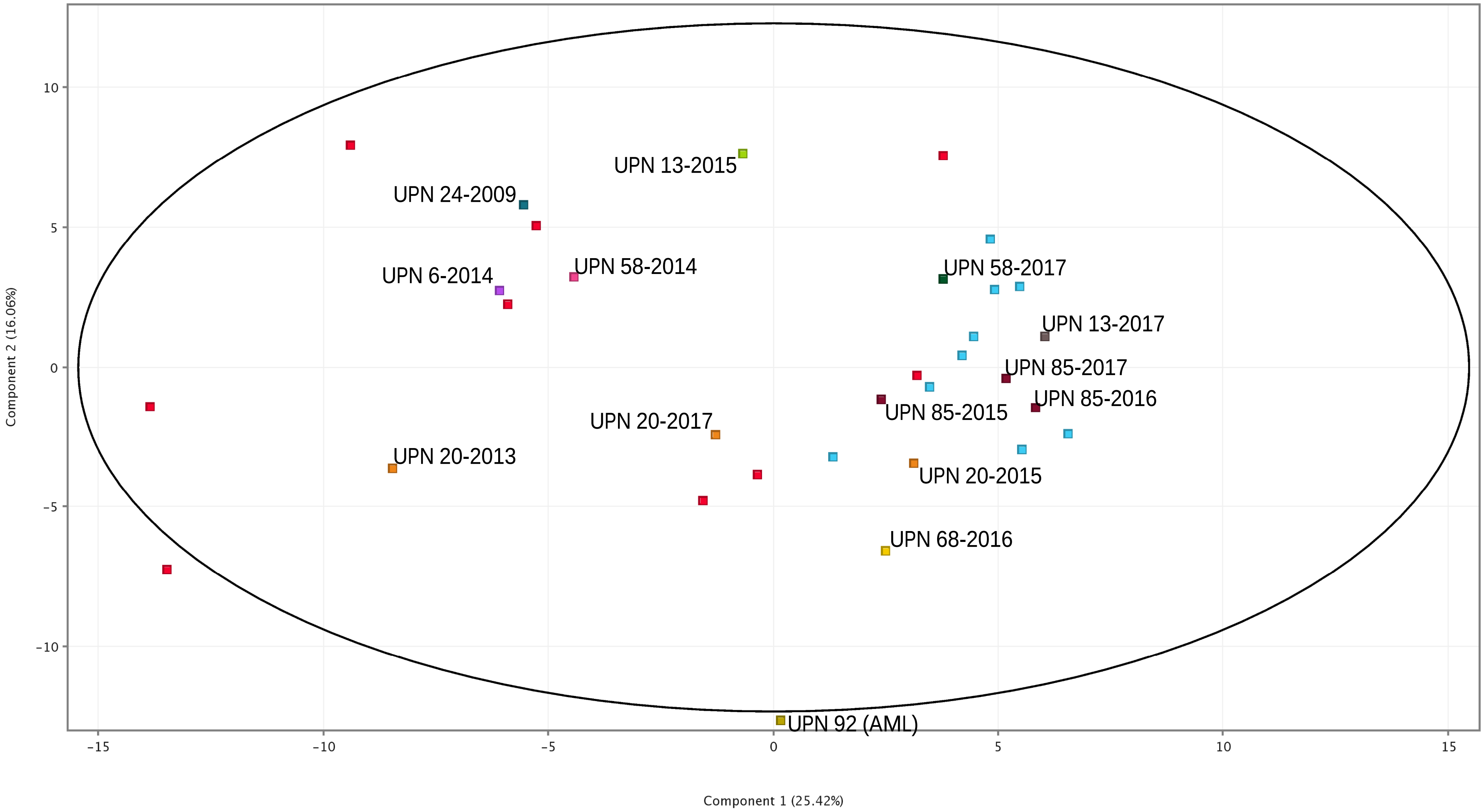

Supplemental Figure 5: Gene Onthology Myeloid Leukocyte Differentiation

Supplemental Fig. 5 Principal Component Analysis (PCA) plot for the Gene set 3 (Gene Ontology Myeloid Leukocyte Differentiation). Healthy controls, SDS-NK patients, and patients with chromosome anomalies are identified as in Fig. 2 and in the text. Component 1 and 2 are indicated in the plot. The 95% confidence interval is shown by a black ellipse.

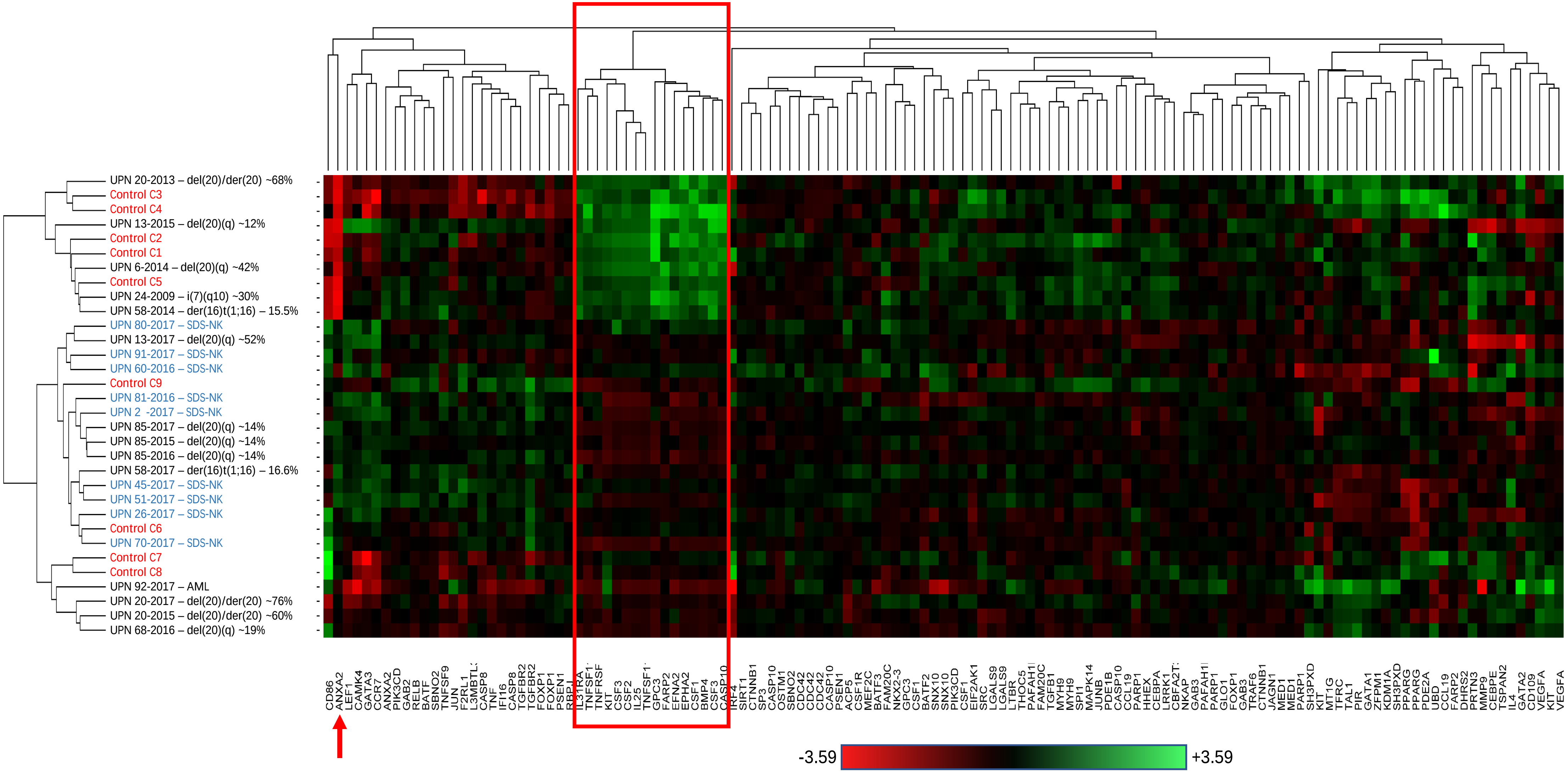

Supplemental Figure 6: Gene Onthology Myeloid Leukocyte Differentiation

Supplemental Fig. 6 Heatmap and dendrogram of cluster analysis on both the axes for the Gene set 3 (Gene Ontology Myeloid Leukocyte Differentiation). Healthy controls, SDS-NK and patients with chromosome anomalies are indicated near the dendrogram lines, with evidence of the sample (year), and the karyotype, as in Fig. 3 in the text. A red arrow and a red box put in evidence genes particularly up/down-regulated, with difference in the groups of controls and SDS-NK. Gene names and $\log_2$ color bar are under the heatmap.
