# Supplementary material for: Microarray expression studies on bone marrow of patients with Shwachman-Diamond syndrome in relation to deletion of the long arm of chromosome 20, other chromosome anomalies or normal karyotype
Source: Mol Cytogenet. 2020 Jan 2;13:1. doi: 10.1186/s13039-019-0466-9 (PMC6941278; doi:10.1186/s13039-019-0466-9)
Supplement: Supplementary file 2 — Additional file 2: Table S1. Blood count and bone marrow cellularity of all the SDS patients here reported at the date of sampling for RNA expression study. [file 13039_2019_466_MOESM2_ESM.pdf]

Table S1: Neutrophil count (x10^3/µl), haemoglobin (Hb) concentration (g/100 ml) and platelet count (x10^3/µl) for the SDS patients here reported. Chromosome clonal anomaly and percentage of abnormal cells are recalled. BM cellularity evaluation is also indicated.

UPN	Sample	Cytogenetics	Neutrophils	Hb	Platelet	BM cellularity
UPN6	2014	del(20)(q11.21q13.13) ~44%	2,39	13,7	57	almost normal
UPN13	2015	del(20)(q11.21q13.32) ~12%	0,63	11	65	mild hypoplasia
UPN13	2017	del(20)(q11.21q13.13) ~52%	1,13	12,5	80	mild hypoplasia
UPN20	2013	del(20)(q11.21q13.32) ~68%	0,5	14,8	91	almost normal
UPN20	2015	del(20)(q11.21q13.32) ~60%	0,3	14,7	73	mild hypoplasia
UPN20	2017	del(20)(q11.21q13.32) ~76%	0,5	14,6	81	mild hypoplasia
UPN58	2014	der(16)t(1;16)(q21;q23) ~17%	0,33	11	134	mild hypoplasia
UPN58	2017	der(16)t(1;16)(q21;q23) ~15%	0,76	10,1	197	mild hypoplasia
UPN68	2016	del(20)(q11.21q13.12)<br>del(20)(q13.12q13.13) ~19%	2,31	17,4	130	mild hypoplasia
UPN85	2015	del(20)(q11.21q11.23) ~14%	0,9	16,5	104	normal
UPN85	2016	del(20)(q11.21q11.23)	0,9	15,9	107	almost normal
UPN85	2017	del(20)(q11.21q11.23) ~11%	2	15,1	96	almost normal
UPN24	2009	i(7)(q10) ~30%	1,9	11,4	108	almost normal
UPN92	2017	complex karyotype[a]	0,54	10,8	12	almost normal[a]
UPN2	2017	normal karyotype	0,7	14,2	113	severe hypoplasia
UPN26	2017	normal karyotype	0,5	13	97	mild hypoplasia
UPN45	2017	normal karyotype	1,08	12	215	severe hypoplasia
PN51	2017	normal karyotype	3,24	12,8	195	almost normal
UPN60	2016	normal karyotype	1,45	11,8	101	normal
UPN70	2017	normal karyotype	1,59	15,4	111	severe hypoplasia
UPN80	2017	normal karyotype	0,32	10,8	174	mild hypoplasia
UPN81	2016	normal karyotype	0,4	11,9	189	not available
UPN91	2017	normal karyotype	1,09	13	193	normal

[a] Patient with Acute Myeloid Leukaemia (AML): data after chemotherapy.
